# Research on the Evolution Law of Electrochemical Impedance Spectral Characteristics of Lithium-Ion Batteries in Different States

**DOI:** 10.3390/molecules31061048

**Published:** 2026-03-22

**Authors:** Xiong Shu, Linkai Tan, Wenxian Yang, Konlayutt Punyawudho, Quan Bai, Qiong Wang

**Affiliations:** 1 Hunan Provincial Key Laboratory of Vehicle Power and Transmission System, Hunan Engineering University, Xiangtan 411104, China; 2 School of Computing and Engineering, University of Huddersfield, Huddersfield HD1 3DH, UK; 3 Department of Mechanical Engineering, Chiang Mai University, Chiang Mai 50200, Thailand

**Keywords:** lithium-ion battery, electric vehicle, EIS, failure characteristics

## Abstract

Lithium-ion batteries (LIBs) are pivotal for energy storage in electric vehicles and renewable systems, but how to effectively monitor their conditions and ensure their operational reliability is still a concern today. This study employs electrochemical impedance spectroscopy (EIS) to systematically investigate the evolution of impedance characteristics in nickel–cobalt–manganese oxide (NCM) lithium-ion batteries (LIBs) under varying states of charge (SOCs), states of health (SOHs), temperatures, and mechanical compression displacements. Results reveal that higher SOC and temperature reduce impedance by enhancing ion kinetics and interfacial activity, with $R_{ct}$ (charge transfer resistance) exhibiting a U-shaped dependence on SOC, minimized at 40–60%. As SOH declines from 100% to 80%, $R_{SEI}$ (SEI film resistance) and $R_{ct}$ increase progressively, reflecting SEI thickening and electrode degradation. Mechanical compression (0–8 mm) elevates all resistances, particularly $R_{ct}$ at high SOC, due to structural deformation and hindered diffusion. DRT (distribution of relaxation times) spectra highlight amplified low-frequency peaks with aging and low SOC, underscoring diffusion limitations. These findings elucidate multi-scale failure mechanisms, from interfacial polarization to structural instability, providing a framework for non-invasive health monitoring and lifetime prediction.

## 1. Introduction

With the global transition in energy structures and the rapid development of renewable energy, lithium-ion batteries (LIBs) have become the core energy storage devices in portable electronic products, electric vehicles [1,2], and energy storage systems due to their long cycle life, excellent safety performance, etc. [3]. However, during long-term use, LIBs inevitably experience abusive conditions such as temperature shock, mechanical compression, and vibration, which lead to capacity fade acceleration and internal shorting, eventually resulting in battery failure. These issues severely limit the reliability of LIBs in practical applications [4,5]. Nevertheless, how such abusive conditions affect the performance characteristics of LIBs remains insufficiently studied. Therefore, investigating the electrochemical impedance characteristics and their evolution under different failure conditions is of great significance for battery performance optimization, remaining use life prediction, and the development of new battery materials.

In real-scenario engineering applications, LIBs may be subjected to mechanical compression [6,7], charging and discharging [8,9], as well as different operation temperature [10,11], which may cause them to be in different working states. To explore the performance characteristics of LIBs under various conditions, researchers have conducted extensive studies. For example, Huang et al. [12] studied the changes in cycling aging performance under compression and found that moderate mechanical compression can improve the cycling stability of batteries. However, when the compression displacement exceeds 6.5 mm, this enhancement effect diminishes and may even accelerate battery degradation. Huang et al. [13] investigated the effects of impact loading on battery failure mechanisms and revealed that conical punch impact causes the most severe damage, while flat punch impact poses potential safety risks. Liu et al. [14] explored the safety performance of LIBs under combined aging and mechanical stress; the results show that the bending resistance of batteries increases with capacity degradation. An et al. [15] analyzed the electrical and thermal behaviors of LIBs under different external short-circuit currents and found that the temperature rise rate is significantly correlated with the short-circuit current and initial state of charge (SOC). Fang et al. [16] performed cryogenic inerting treatments on spent LIBs using liquid nitrogen (LN) and dry ice (DI), and evaluated their thermal stability. The results showed that low-temperature conditions led to electrolyte solidification, component separation, and increased internal resistance. Qu et al. [17] studied the force–electro-thermal responses of aged LIBs under mechanical abuse and found that, while the mechanical properties change after aging, the electrical characteristics remain relatively stable. Ren et al. [18] investigated the performance of commercial LIBs under different overcharge conditions and found that electrolyte decomposition and metal dissolution occurred at the cathode, while severe lithium plating was observed at the anode. Liu et al. [19] examined the capacity fade mechanisms under various discharge rates, revealing a positive correlation between discharge rate and degradation. The results show that, after 4000 cycles at 5C, 10C, and 20C, the capacity retention rates were 75%, 79.1%, and 64.8%, respectively. Han et al. [20] studied the capacity loss mechanisms of commercial cylindrical LIBs under high dynamic mechanical impacts. Zhou et al. [21] investigated the thermal behavior and failure mechanisms of pouch cells under high-rate overdischarge conditions. Xu et al. [22] established a failure assessment method for cylindrical LIBs based on rigid punch impact tests at different loading rates. The results showed that the thermal runaway safety boundary (TCSB) and ultimate critical safety boundary (UCSB) decreased logarithmically with increasing loading rate. Zhou et al. [23] revealed the triggering mechanism of thermal runaway in LIBs. Zhao et al. [24] explored the relationship between jelly roll structural failure and internal short circuits (ISCs) in LIBs under mechanical loading.

Apart from that, Spielbauer et al. [25] analyzed the failure mechanisms of 18650 LIBs under dynamic mechanical loading. Zhang et al. [26] combined experimental methods with finite element modeling to systematically investigate the dynamic response and failure mechanisms of pouch-type LIBs under impact loading. The results indicated that impact energy, loading rate, and punch geometry are the key factors influencing battery failure. Zhou [27] explored the failure mechanisms of commercial pouch LIBs within a temperature range of −30 °C to 70 °C. The study showed that localized damage to the separator at low temperatures could easily trigger early battery failure, while high temperatures could lead to internal short circuits and structural damage of the cell. Liu et al. [28] investigated the effects of mechanical compression on battery capacity and cycling performance. Chen et al. [29] studied the failure mechanisms of 18650 battery packs and concluded that prolonged overheating results in active material delamination and dense stacking of separator pores, eventually causing thermal runaway. Mao et al. [30] conducted a series of nail penetration tests to analyze the influence of SOC, penetration location, depth, and speed on the thermal runaway behavior of LIBs. The results showed that center penetration is more likely to induce severe thermal runaway. Wang et al. [31] investigated the effects of slight deformation on battery degradation behavior and found that even a 3.1% deformation could cause an instantaneous capacity drop of up to 6.3%. Zhang et al. [32] assessed the performance evolution of LIBs under different aging paths through overcharge experiments, and the results indicated that overcharge tolerance deteriorates with battery aging, and performance degradation is most significant under low-temperature and high-rate cycling conditions. Zeng et al. [33] examined the external short-circuit characteristics and thermal runaway behavior of 18650-type NCM LIBs under different SOC levels and short-circuit currents. Wang et al. [34] investigated the electrochemical degradation mechanisms of LIBs under room temperature and high-temperature cycling conditions; the findings revealed that low temperatures exacerbate localized lithium plating, leading to rapid capacity fade, internal short circuits, and separator melting. Shu [35] discussed the effects of temperature, SOC, and compressive deformation on the mechanical, electrical, and thermal properties of LiFePO_4_ batteries.

Although the above studies have provided valuable insights into the failure characteristics of LIBs under different operating or abuse conditions, most of them have focused on individual aspects such as structural deformation, OCV response, thermal behavior, or microscopic morphology after damage. In comparison, systematic investigations of how the impedance response evolves in one unified NCM cell system under multiple state variables—including temperature, SOC, SOH, and mechanical compression—remain relatively limited. In addition, many previous EIS-related studies have emphasized impedance spectra themselves, while fewer works have combined EIS with equivalent circuit fitting and DRT analysis to compare interfacial, charge transfer, and diffusion-related contributions across different battery states in a unified framework.

To address these gaps, this study investigates the evolution of electrochemical impedance characteristics of commercial 18650 NCM lithium-ion batteries under four representative state dimensions, namely temperature, SOC, SOH, and mechanical compression displacement. The novelty of this work lies in three aspects. First, the impedance evolution under these different operating and degradation states is examined within one consistent NCM cell system and test framework, enabling direct cross-condition comparison. Second, EIS, ECM fitting, and DRT analysis are combined to distinguish the variations in ohmic resistance, interfacial resistance, charge transfer behavior, and diffusion-related response. Third, the work establishes a comparatively systematic impedance-based description of battery state evolution, which is useful for non-invasive diagnosis and health-state assessment under multi-condition scenarios.

## 2. Experimental Method

### 2.1. Test Equipment

**Figure 1:**
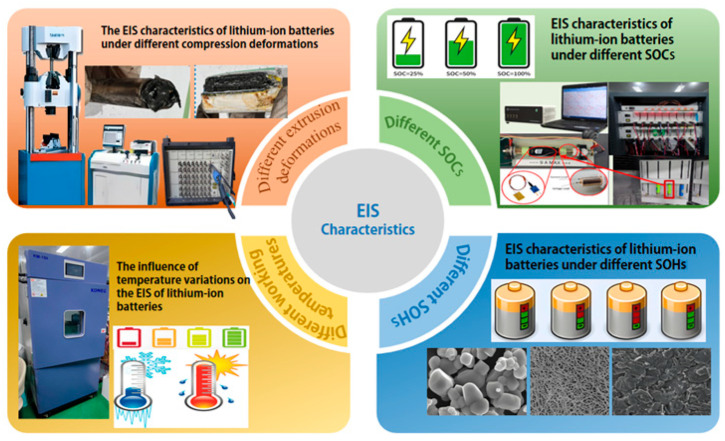
Experimental setup.

To investigate the electrochemical impedance characteristics and failure behavior under compressive deformation, commercially available 18650 NCM lithium-ion cells were selected in this study. The nominal capacity of the cells was 1500 mAh. According to the available product specification, the cathode material was NCM, the anode material was graphite, the separator was a polyolefin film, and the electrolyte salt was LiPF6. However, the detailed electrolyte solvent composition was not disclosed by the manufacturer. This point has been acknowledged as a limitation of the present study. Nevertheless, all experiments were conducted using the same cell type, which ensures the internal consistency of the comparative analysis under different operating conditions. The experimental setup and procedure are illustrated in Figure 1.

### 2.2. Test Method

**Figure 2:**
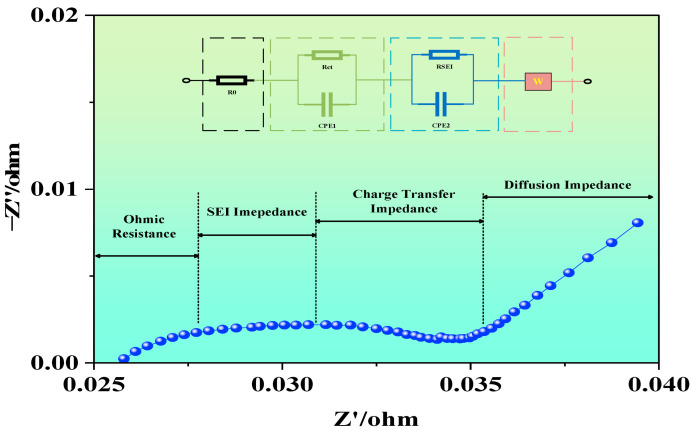
Equivalent circuit model and Nyquist plot.

EIS is a non-destructive, high-precision, and high-resolution electrochemical characterization technique. By fitting the measured impedance modulus, imaginary part, and phase angle, and combining the DRT method, one can obtain the relaxation time distribution and parameter variation trends corresponding to each impedance process. At present, the most commonly used analytical method for EIS is the equivalent circuit approach, which empirically decomposes the internal electrochemical impedance of the battery into multiple RC circuits. Relevant software (ZView3.3) is then used to fit and extract the impedance values of the circuit components. In theory, due to the numerous side reactions within LIBs, an infinite number of RC loops could be used to describe different internal resistive behaviors. However, due to the high computational load of such fitting, simplification is required in practice. In this study, a commonly used second-order equivalent circuit model is applied to analyze the EIS evolution characteristics of LIBs under various conditions, as illustrated in Figure 2.

To avoid confusion regarding the different test matrices used in this work, it should be noted that the SOC–temperature conditions were intentionally selected according to the objective of each experimental stage. For the fresh-cell EIS characterization, a relatively wide SOC range (5%, 20%, 50%, 70%, and 90%) and temperature range (15 °C, 25 °C, and 35 °C) were adopted to establish the baseline effects of operating conditions on impedance behavior. For the SOH-related analysis, the tests were restricted to representative intermediate SOC levels (50% and 70%) and moderate temperatures (15 °C and 25 °C) to better isolate the influence of aging while reducing the interference of extreme operating conditions. For the mechanical compression experiments, the temperature was fixed at 25 °C and three representative SOC levels (0%, 50%, and 90%) were selected to evaluate the effect of compression under low, medium, and high charge states. Therefore, the different parameter spaces used in the three parts were purposefully designed based on different research aims.

For the SOH-related analysis, the cells with different SOH levels were obtained through controlled cycling aging. In this study, SOH was defined based on capacity retention relative to the initial discharge capacity of the fresh cells, i.e.,

(1) $$SOH=\frac{C_{aged}}{C_{fresh}}\times 100\%$$

where $C_{aged}$ is the measured discharge capacity of the aged cell and $C_{fresh}$ is the initial discharge capacity of the fresh reference cell. Before the aging tests, all fresh cells were subjected to consistency screening, including capacity and internal resistance measurement. The selected cells with stable initial performance were treated as the 100% SOH reference group. The aged cells corresponding to different SOH levels were then obtained through repeated charge–discharge cycling, and their SOH values were determined by periodic capacity measurement until the target capacity retention levels were reached.

During EIS testing, the impedance spectra were recorded on a CHI700E electrochemical workstation (Shanghai Chenhua Instrument Co., Ltd., Shanghai, China). Prior to each measurement, the cells were rested until the OCV stabilized (≥30 min). The EIS measurements were then carried out at OCP, using a 10 mV rms sinusoidal perturbation over the frequency range from 10 kHz to 1 mHz. To ensure fitting quality and measurement reliability, each spectrum contained at least six points per decade on a logarithmic frequency axis, and open/short cable compensation was performed prior to measurements. To ensure measurement repeatability, the EIS test under each condition was repeated 2–3 times after stabilization. The repeated spectra showed generally consistent overall trends. The spectra presented in the figures are representative individual curves selected from repeated measurements with similar overall trends. Current studies show that the characteristic frequency range of the SEI film impedance peak is approximately 36–76 Hz, 10–1000 Hz, 16–168 Hz. The anode–electrolyte interface charge transfer impedance peak appears in 0.01–168 Hz, and the cathode–electrolyte interface charge transfer impedance peak appears in 0.4–3 Hz, 2–14 Hz. The solid-phase diffusion impedance peak typically appears below 10 Hz. Thus, when analyzing impedance spectra, one can distinguish between processes based on the features of high-, medium-, and low-frequency regions.

The high-frequency region primarily reflects the ohmic resistance associated with ion transport through the electrolyte, porous materials, active material particles, and current collectors. In the mid-frequency region, the EIS spectrum typically exhibits one or more incomplete semicircles, known as capacitive arcs. These arcs represent the participation of electrons and lithium ions in the charge transfer process at the electrode–electrolyte interface and indicate the interfacial charge transfer resistance. In the low-frequency region, the EIS spectrum is usually characterized by a diagonal line, which corresponds to the diffusion process of ions from the electrolyte to the electrode surface or from the electrode surface into the bulk electrode. The main influencing factors include the crystal structure, particle size, and porosity of the electrode materials, as well as operating conditions and temperature.

During EIS testing, the AC voltage and response current can be expressed as follows:

(2) $$E(t)=E_{0}\sin(\omega t),I(t)=I_{0}\sin(\omega t+\theta )$$

So, the complex impedance at a given frequency is defined as follows:

(3) $$Z(\omega )=\frac{E(\omega )}{I(\omega )}=Z_{\mathrm{re}}+jZ_{\mathrm{im}}$$

where $Z_{\mathrm{re}}$ represents the real part (resistive behavior), and $Z_{\mathrm{im}}$ represents the imaginary part (capacitive, inductive, or diffusive behavior).

The impedance modulus and phase angle are given by the following:

(4) $$|Z(\omega )|=\sqrt{Z_{\mathrm{re}}^{2}+Z_{\mathrm{im}}^{2}}\theta (\omega )={tan}^{-1}\left(\frac{-Z_{\mathrm{im}}}{Z_{\mathrm{re}}}\right)$$

The typical impedance response of an LIB can be described using the Randles equivalent circuit, which consists of a series resistance $R_{0}$, charge transfer resistance $R_{ct}$, and double-layer capacitance $C_{dl}$, yielding a complex impedance:

(5) $$Z(\omega )=R_{0}+\frac{R_{ct}}{1+j\omega R_{ct}C_{dl}}$$

Expanded into real and imaginary components:

(6) $$Z(\omega )=R_{0}+\frac{R_{ct}}{1+\omega^{2}R_{ct}^{2}C_{dl}^{2}}-j\frac{\omega C_{dl}R_{ct}^{2}}{1+\omega^{2}R_{ct}^{2}C_{dl}^{2}}$$

In the low-frequency region, the diffusion effect at the electrode/electrolyte interface introduces Warburg impedance, expressed as follows:

(7) $$Z_{w}(\omega )=\sigma (1-j)\omega^{-1/2}$$

where σ is the Warburg coefficient, reflecting diffusion resistance. Under semi-infinite diffusion conditions, this contribution gives rise to an approximately 45° sloped line in the Nyquist plot. In practical battery electrodes, however, diffusion is often finite-length because of bounded particle geometry and limited transport distance, so the low-frequency response may gradually deviate from the ideal 45° line and evolve toward a more vertical trend.

In practical applications, the behavior of the double-layer capacitance often deviates from ideality, requiring a constant phase element (CPE) for accurate representation:

(8) $$Z_{CPE}=\frac{1}{Q(j\omega {)}^{n}}$$

where Q is the CPE parameter, and n∈(0,1) is the phase angle correction factor, reflecting surface roughness or interface heterogeneity.

To overcome the structural limitations of predefined equivalent circuits, this study also adopts the DRT method to deconvolve the impedance response. In this method, the system impedance is expressed as the superposition of multiple relaxation processes

(9) $$Z(\omega )=R_{s}+\int_{0}^{\infty }\frac{\gamma (\tau )}{1+j\omega \tau }d\ln\tau$$

where γ(τ) is the relaxation time distribution function (i.e., the DRT spectrum), which is obtained via deconvolution of $Z_{\mathrm{im}}(\omega )$. This approach enables the separation and identification of interfacial reactions, charge transfer, and diffusion processes, thereby enhancing the resolution and reliability of internal battery process analysis.

By analyzing the impedance modulus, phase angle, and DRT spectra of LIBs under various conditions, the mapping relationship between different failure modes and EIS characteristics can be derived. This provides a theoretical basis for battery state monitoring and health management.

## 3. Results and Analysis

Based on the testing methods and equipment described above, we conducted a study on the evolution of impedance characteristics under different SOCs, SOHs, temperatures, and mechanical deformation levels. The detailed results are presented as follows.

### 3.1. EIS Characteristics Under Different SOCs

**Figure 3:**
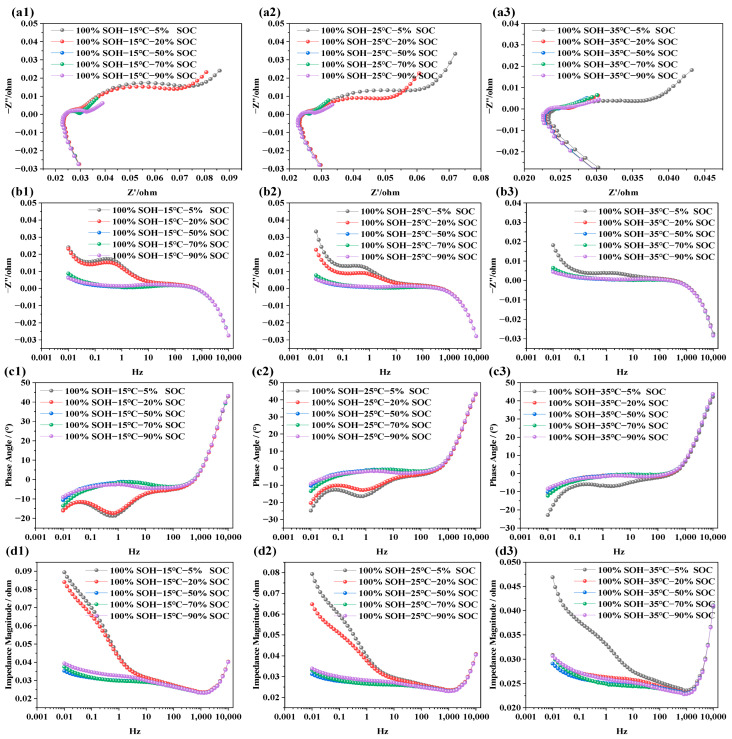
Impedance spectra of LIBs under different SOC conditions: (**a1**–**a3**) show the EIS at 15 °C, 25 °C, and 35 °C under various SOC levels; (**b1**–**b3**) display the imaginary part of impedance at different SOC levels and temperatures; (**c1**–**c3**) illustrate the phase angle variations under different SOC levels; (**d1**–**d3**) present the impedance modulus across frequencies for different SOC levels.

The purpose of testing the impedance characteristics of LIBs under different SOCs is to explore the internal electrochemical behavior and its mapping relationship with capacity levels. In this study, refreshment NCM LIBs were selected. Tests were carried out at SOC levels of 5%, 20%, 50%, 70%, and 90%, under three different temperatures: 15 °C, 25 °C, and 35 °C. This was done to systematically investigate the influence of SOC on electrochemical impedance behavior. The test results are shown in Figure 3.

In Figure 3a1–a3, the EIS of LIBs with SOCs of 5%, 20%, 50%, 70%, and 90% are displayed across a frequency range of 0.01 Hz to 10 kHz at temperatures of 15 °C, 25 °C, and 35 °C, respectively. It is evident that batteries with higher SOCs show smaller impedance semicircles, while lower SOCs correspond to larger semicircles. This trend is not due to a change in the total lithium content within the cell, but rather to the redistribution of lithium between the electrodes and the corresponding variation in electrode lithiation state at different SOC levels. Under lower-SOC conditions, the electrode reaction kinetics are more constrained and the charge transfer resistance is higher, leading to a larger impedance semicircle. In contrast, under higher-SOC conditions, the interfacial electrochemical processes become more favorable, resulting in lower impedance. This trend remains consistent across all three temperature conditions. Figure 3b1–b3 is the frequency response of the imaginary part of the impedance; from the figure, we can see that, in the low-frequency domain, the imaginary part is positive with capacitive lag. As the frequency increases, the imaginary part drops rapidly and may become negative. Once the frequency exceeds 10 Hz, the SOC has negligible influence on the imaginary part. This indicates that the ohmic resistance in the high-frequency region, associated with electrolyte and current collectors, is independent of SOC, while the polarization resistance, charge transfer, and diffusion behaviors in the mid-to-low-frequency regions are closely tied to SOC. The phase angle in EIS represents the phase shift between voltage and current and reflects the relative contributions of resistive and capacitive responses. In Figure 3c1–c3, the phase angle is negative in the frequency range of 0.001 Hz to 10 Hz, indicating dominant capacitive behavior. As the scanning frequency increases, the phase angle gradually approaches zero. Between 10 Hz and 100 Hz, the response becomes more resistive. Beyond 1000 Hz, the phase response shows characteristics associated with parasitic inductance in the measurement circuit. As SOC increases, the phase angle becomes flatter in the mid-to-low-frequency region, implying faster charge transfer and reduced capacitive lag within the battery. Notably, at 15 °C and 25 °C, batteries with lower SOC levels (e.g., SOC = 5%, 20%) exhibit more pronounced capacitive behavior. In Figure 3d1–d3, the total impedance modulus across frequencies is shown. The overall impedance decreases with increasing frequency. However, when the frequency exceeds 1000 Hz, the total impedance begins to rise sharply. Batteries at lower SOC levels show higher total impedance, while those at higher SOCs exhibit relatively lower impedance. This is primarily due to the dominance of capacitive impedance in the low-frequency region and resistive impedance in the high-frequency region. At low SOC, the battery exhibits slower interfacial charge transfer kinetics and higher solid-state/electrolyte diffusion resistance, which together result in a higher overall impedance response.

### 3.2. EIS Characteristics Under Different Temperatures

**Figure 4:**
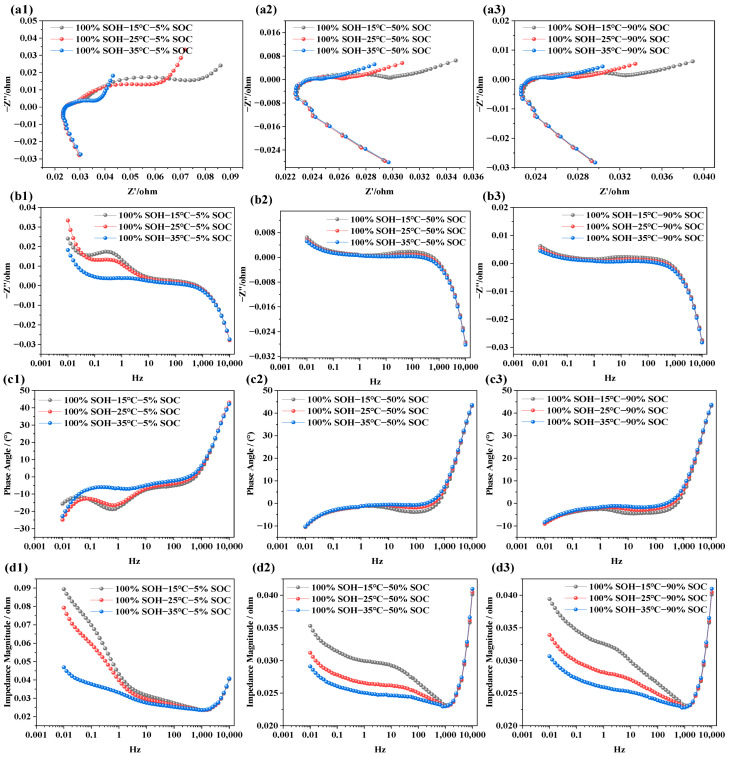
Impedance spectra of LIBs at different temperatures: (**a1**–**a3**) EIS at SOC levels of 5%, 50%, and 90% under various temperatures; (**b1**–**b3**) imaginary part of impedance at different temperatures and SOC levels; (**c1**–**c3**) phase angle variation at different temperatures and SOC levels; (**d1**–**d3**) impedance modulus variation under different temperatures and SOC conditions.

The objective of analyzing the impedance characteristics of LIBs under different temperature conditions is to reveal how temperature variations affect internal electrochemical responses and degradation mechanisms. Similar to the EIS tests under different SOC conditions, the NCM LIBs with SOH = 100% were selected for testing. Measurements were carried out at three temperature levels (i.e., 15 °C, 25 °C, and 35 °C). During each temperature test, the batteries were set to three SOC states—5% (i.e., low state of charge), 50% (i.e., nominal state), and 90% (fully charged)—in order to systematically investigate the influence of temperature on battery electrochemical impedance behavior. The test results are shown in Figure 4.

From Figure 4a1–a3, it can be observed that the impedance semicircle decreases with rising temperature. This indicates that ionic conductivity in the electrolyte improves with temperature, facilitating faster ion transport and easier ion exchange at the electrode–electrolyte interface, thereby reducing interfacial polarization. This effect is more pronounced under low-SOC conditions, whereas at high SOC, due to the near-saturation of lithium ions, the influence of temperature becomes less significant.

In Figure 4b1–b3, it is evident that, at SOC = 5%, temperature significantly affects the imaginary part of the impedance in the low-frequency region (i.e., 0.01–10 Hz). However, as SOC increases (i.e., SOC = 50%, 90%), the variation in the imaginary component with temperature in this frequency range becomes negligible. This suggests that, at low SOC, ion diffusion and charge transfer are the primary limiting factors, and increasing temperature accelerates these processes. In contrast, at high SOC, internal reactions are already completer and more stable, reducing the sensitivity of the imaginary component to temperature. Nonetheless, due to other factors such as internal resistance and conductivity, temperature still affects the impedance modulus. In Figure 4c1, the phase angle shows a clear shift with temperature, especially within the 0.1–10 K Hz range, where the phase angle peak moves from −30° to −5° as temperature increases. However, in Figure 4c2,c3, the phase angle curves under different temperatures are almost identical, indicating minimal influence. This suggests that, under low SOC, the charge transfer and lithium-ion diffusion kinetics are more strongly constrained, and temperature therefore plays a more significant role in facilitating interfacial electrochemical processes. In contrast, under high-SOC conditions, the interfacial charge transfer and ion-transport processes become less rate-limiting, so the impedance response exhibits lower sensitivity to further temperature increase. In Figure 4d1–d3, the effect of temperature on total impedance is illustrated. As temperature increases, the total impedance in the low-frequency region decreases significantly under low SOC. In contrast, under high SOC, both low- and mid-frequency impedance values decrease notably. This may be because, at low SOC, the limited number of active lithium ions makes the electrode reactions more dependent on interfacial processes—primarily charge transfer. Since temperature increase enhances interfacial reaction rates, it reduces charge transfer resistance, which is most apparent in the mid-to-low frequency range. Additionally, batteries are more polarized at low SOC, with reaction limitations concentrated in the low-frequency region, leading to a noticeable impedance drop there. At high SOC, the interfacial electrochemical processes become less rate-limiting, and the temperature sensitivity of the impedance response is therefore reduced. In contrast, the diffusion-related contribution remains more pronounced under low-SOC conditions, which is reflected by the larger low-frequency response.

### 3.3. EIS Characteristics Under Different SOH Conditions

**Figure 5:**
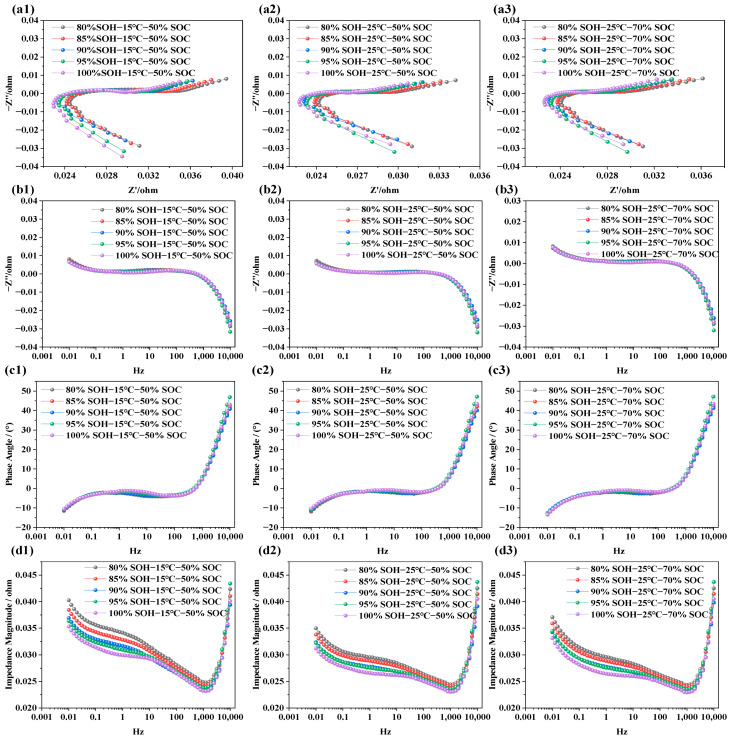
Electrochemical impedance spectra of LIBs under different SOH conditions. (**a1**–**a3**) EIS at 15 °C, and 25 °C under various SOHs; (**b1**–**b3**) imaginary part of impedance at 15 °C, and 25 °C under various SOHs; (**c1**–**c3**) phase angle variation at 15 °C and 25 °C under various SOHs; (**d1**–**d3**) impedance modulus variation at 15 °C and 25 °C under various SOHs.

The impedance characteristics of LIBs under different SOHs aim to reveal the impact of capacity degradation on internal electrochemical behavior. Similar to the EIS tests conducted under varying SOC and temperature conditions, NCM LIBs with SOH values of 100%, 95%, 90%, 85%, and 80% were selected as test subjects. Additionally, EIS measurements were performed under two temperature conditions (i.e., 15 °C and 25 °C) and two SOC levels (i.e., 50% and 70%) to further analyze and compare the effects of capacity degradation on electrochemical impedance characteristics. The results are presented in Figure 5.

From Figure 5a1–a3, it is observed that, as SOH decreases (i.e., as battery capacity degrades), the overall EIS curve shifts noticeably to the right. This trend is evident under the tested conditions of 15 °C and 25 °C and at SOC = 50% and 70%, suggesting that capacity fade is accompanied by an increase in ohmic resistance. Within this investigated mid-SOC range, the dependence of ohmic resistance on SOC appears relatively weak. In Figure 5b1–b3, the extracted frequency vs. imaginary part curves shows that, within the frequency range of 0.001 Hz to 10 kHz, the influence of SOH on the imaginary impedance is not uniform across the frequency range. The variation is relatively limited in the high-frequency region, whereas the low-frequency diffusion-related response becomes more pronounced with decreasing SOH. A similar trend is observed in the phase angle plots (i.e., Figure 5b1–b3), suggesting that changes in SOH do not cause significant shifts in phase angle behavior. However, as the frequency increases, the phase angle rises noticeably in the high-frequency region, reflecting the influence of parasitic inductance from the test leads, fixtures, and connection loop. In Figure 5d1–d3, the total impedance plots reveal a typical trend: As frequency increases, the total impedance magnitude decreases at first, but rises rapidly after 1000 Hz. Under low SOC, total impedance values are consistently higher, while higher-SOC conditions correspond to lower total impedance values. This is primarily because, in the low-frequency region, the capacitance characteristics of the battery play a dominant role, whereas in the high-frequency region, resistive (ohmic) characteristics of the battery become more significant. Once frequency exceeds 4500 Hz, the upward trend of the impedance response is dominated by parasitic inductive effects in the measurement setup. Moreover, at low SOC, internal reactions become more limited, and diffusion resistance increases, resulting in overall higher impedance.

### 3.4. EIS Characteristics Under Different Compression Displacements

**Figure 6:**
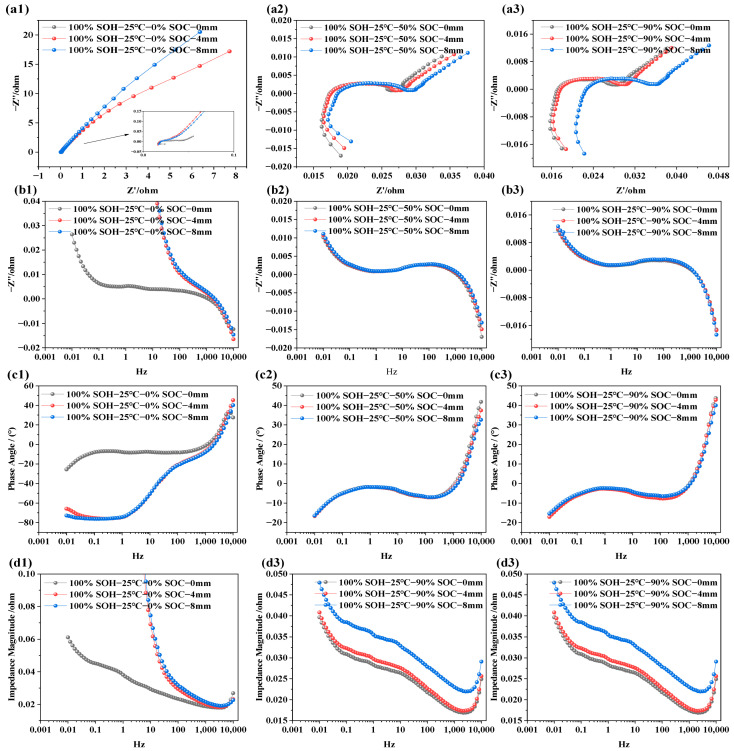
EIS feature of LIBs under different compression displacements; (**a1**–**a3**) EIS at SOC is of 5%, 50%, and 90% under various extrusion displacements; (**b1**–**b3**) imaginary part of impedance at different extrusion displacements and different SOCs; (**c1**–**c3**) phase angle variation at different extrusion displacements and different SOCs; (**d1**–**d3**) impedance modulus variation at different extrusion displacements and different SOCs.

Unlike external or cycling conditions such as temperature, SOC, and SOH, mechanical compression causes irreversible microstructural deformation within the battery. This physical compaction process not only affects the porosity and overall deformation of the electrode materials but may also lead to the destruction or rearrangement of interfacial structures. However, during battery module assembly or usage, varying degrees of compression deformation are inevitable. To comprehensively assess the impact of mechanical deformation on the electrochemical performance of LIBs, this study investigates the evolution of electrochemical impedance characteristics under three different compression displacements (i.e., 0 mm, 4 mm, and 8 mm). This investigation not only contributes to understanding battery failure mechanisms, but also provides valuable data for structural optimization and safety enhancement. The test results are shown in Figure 6.

Figure 6 displays the EIS of LIBs at 25 °C with SOC levels of 0%, 50%, and 90%, measured over a frequency range of 0.01 Hz to 10 kHz. From Figure 6a1–a3, it can be observed that SOC has a relatively minor influence on ohmic resistance. As compression displacement increases, the ohmic resistance slightly increases under all SOC conditions. This is primarily attributed to the compression-induced densification of the porous electrodes and separator, which reduces porosity and increases tortuosity in the electrolyte-filled ion-transport pathways, thereby lowering the effective ionic conductivity and slightly increasing the ohmic resistance. Additionally, mechanical pressure may cause minor structural damage, potentially leading to electrolyte leakage or decomposition, further obstructing ion conductivity and increasing ohmic resistance. Furthermore, the results from Figure 6b1–b3,c1–c3 show that, at low SOC (i.e., SOC = 0%), the capacitive characteristics of the battery are significantly enhanced. However, this effect diminishes as SOC increases. At SOC = 50% and 90%, the capacitive characteristics remains nearly unchanged after compression from 0 to 8 mm. Apart from that, Figure 6d1–d3 demonstrates that, across different SOC levels, mechanical compression leads to an increase in total impedance. This may be attributed to the physical pressure densifying the electrode materials, which alters the pore structure and reduces porosity, thereby impeding lithium-ion diffusion and transport within the electrode. Reduced porosity results in longer diffusion paths and hence higher diffusion impedance. The compression-induced densification of the electrode structure is therefore a key factor in performance degradation under mechanical stress.

### 3.5. DRT Analysis

**Figure 7:**
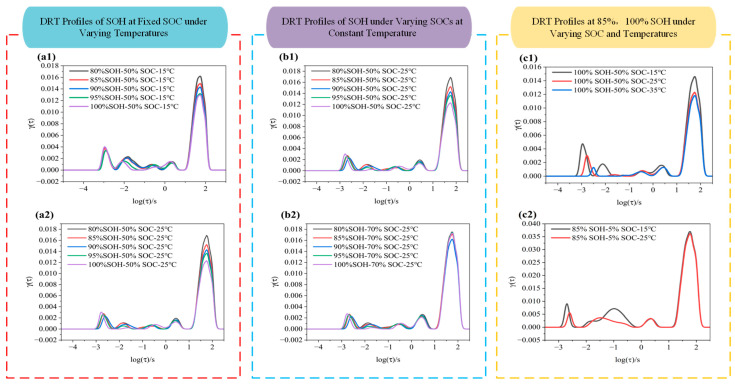
DRT curves of LIBs under different conditions: (**a1**,**a2**) DRT profile at 15 °C and 25 °C under various SOHs; (**b1**,**b2**) DRT profile at SOC 50% and 70% under various SOHs; (**c1**,**c2**) DRT profile at SOH 100% and 85% under various SOCs.

The DRT is a mathematical approach used to deconvolve EIS data and extract internal electrochemical processes within a battery. By analyzing complex impedance responses, DRT can identify characteristic features of various processes such as ion diffusion in the electrolyte, charge transfer, and interfacial resistance. To further investigate the impedance characteristics of LIBs under varying SOHs, SOCs, and temperatures, this section presents DRT analysis for different states of the battery. The outcomes are illustrated in Figure 7.

As shown in Figure 7, the DRT profiles across different frequency ranges exhibit distinct peak features. For instance, in Figure 7a1, within the log(τ/s) ≈ 2.2 to 1 range (i.e., corresponding to the low-frequency region), all curves display a major peak with a height of approximately 0.013. As SOH decreases from 100% to 80%, the peak height slightly increases (from ~0.013 to ~0.016), indicating that battery degradation leads to higher diffusion impedance. In the mid-to-high-frequency region (log(τ/s) = 1 to –2), the curves are smooth and show no significant diffusion peaks. When the temperature increases to 25 °C (as shown in Figure 7a2), the enhancement of peak height with SOH degradation becomes more pronounced (e.g., SOH 80% shows a peak height of ~0.017, higher than 0.013 for SOH 100%). Additionally, the peak differentiation between different SOH levels becomes more visible, suggesting that higher temperature amplifies the impact of degradation on charge transfer processes. The mid-to-high-frequency region remains flat with no notable features, indicating that degradation effects are more discernible at 25 °C. In Figure 7b2, when SOC is raised to 70%, the main peak height slightly decreases, but SOH degradation still causes an increase in peak height (e.g., ~0.017 at SOH 80%). Moreover, a shoulder peak appears around log(τ/s) ≈ 0 to 1, indicating that a higher SOC introduces a slight diffusion component. Overall, the DRT curves under these conditions are smoother, implying that degradation mainly affects the mid-frequency region, and that higher SOC may suppress certain high-frequency impedance contributions.

By comparing Figure 7c1 and Figure 7c2, it is observed that, when SOC = 50%, the main peak height ranges from ~0.012 to 0.015, while at SOC = 5%, the DRT spectrum becomes more complex with multiple shoulder peaks. In fresh batteries (i.e., SOH = 100%), low SOC (i.e., SOC = 5%) combined with low temperature (i.e., 15 °C) results in a significantly increased main peak height (~0.036) at log(τ/s) ≈ 1.6. When the temperature rises to 25 °C, the peak becomes lower and smoother, indicating that higher temperature accelerates electrochemical processes, reduces impedance, and eliminates pronounced low-frequency peaks. At low SOC, the DRT spectrum exhibits a higher main peak and more pronounced shoulder features, indicating a stronger diffusion-related contribution and more complex polarization behavior.

### 3.6. Analysis of Impedance Parameters

**Figure 8:**
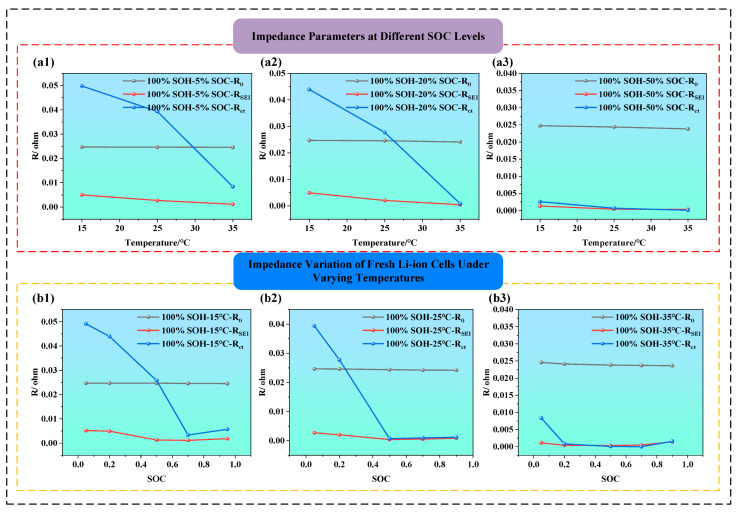
ECM parameter evolution of fresh LIBs under different temperatures and SOCs (**a1**–**a3**) ECM parameter evolution of fresh LIBs under different temperatures; (**b1**–**b3**) ECM parameter evolution of fresh LIBs under different SOCs.

**Figure 9:**
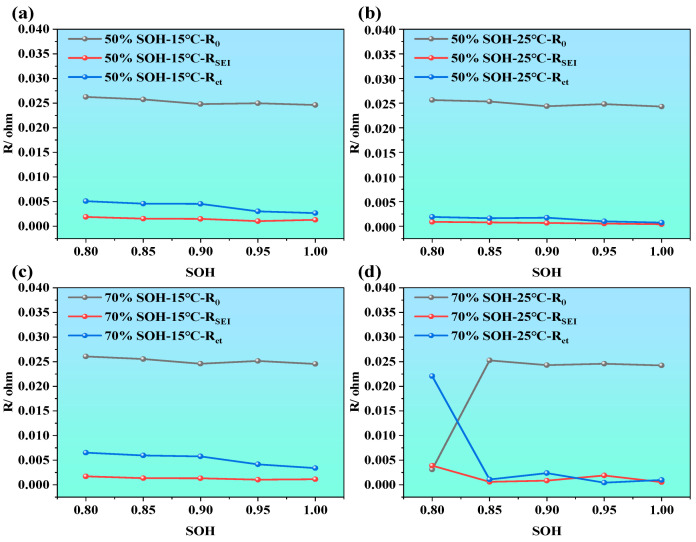
ECM parameter evolution of LIBs during the aging process under different SOC and temperature conditions; (**a**) 15 °C and 50% SOC, (**b**) 25 °C and 50% SOC, (**c**) 15 °C and 70% SOC, and (**d**) 25 °C and 70% SOC.

**Figure 10:**
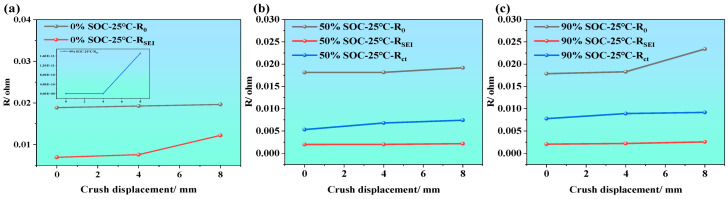
ECM parameter evolution of LIBs under mechanical compressions at different SOCs; (**a**–**c**) ECM parameter evolution of LIBs at SOC = 0%, 50%, and 90% under various crush displacements.

To quantitatively analyze the evolution of impedance parameters during the different conditions of LIBs, this section performs parameter fitting based on the second-order equivalent circuit model. As shown in Figure 2, this circuit typically includes the ohmic resistance (R_0_), SEI film resistance (RSEI), charge transfer resistance (Rct), and a constant phase element (CPE) representing double-layer capacitance in parallel. Based on the nonlinear least squares fitting method, the EIS data was applied to fit the values of each impedance parameter, and the obtained results are presented in Figure 8, Figure 9 and Figure 10.

Figure 8a1–a3 is the effect of temperature on impedance parameters in fresh batteries (i.e., SOH = 100%) at SOC = 5%, 20%, and 50%. It is clear that, as temperature increases from 15 °C to 35 °C, the value of impedance components declines, especially Rct, which shows the most pronounced drop. For example, when SOC is 5% (i.e., as shown in Figure 8a1), the temperature from 15 °C to 35 °C reduces, and the Rct declines by approximately threefold. From the perspective of electrochemical kinetics, this is because, at low SOC, lithium-ion concentration is low and reaction kinetics are sluggish, while higher temperature significantly enhances the activation energy, accelerating interfacial reaction rates. Additionally, the ionic conductivity of the SEI layer improves at higher temperatures, leading to a noticeable reduction in RSEI. At SOC = 50% (i.e., as shown in Figure 8a3), the impact of temperature becomes much less significant, R_0_ and RSEI remain nearly stable, and Rct only shows a slight decrease, suggesting that the battery system is already at electrochemical optimal conditions, where temperature is no longer a limiting factor.

Figure 8b1–b3 illustrate how impedance parameters vary with SOC under different temperature conditions (i.e., the temperature is at 15 °C, 25 °Cand 35 °C). The charge transfer resistance Rct displays a typical U-shaped trend, being higher at low and high SOC, and lower at mid-SOC, indicating maximum electrochemical activity and minimal polarization near 50% SOC. This suggests that both very low and very high SOC can hinder interfacial reactions, thereby increasing Rct. Additionally, RSEI tends to be higher at low SOC, indicating that the SEI film becomes denser under deeper discharge, which restricts lithium-ion migration. Comparatively, both Rct and RSEI are significantly lower at 35 °C than at 15 °C, indicating that higher temperatures enhance the migration rate of lithium ions and electrons, facilitate interfacial reactions, and reduce impedance. The thermal activation thus offsets SOC-induced impedance variation, highlighting the regulatory role of temperature in interfacial kinetics.

As illustrated in Figure 9a–d, under the different temperatures (i.e., 15 °C and 25 °C) and the different SOCs (i.e., 50%, and 70%), all impedance parameters exhibit an upward trend with the decrease in SOH. Among them, the ohmic resistance R_0_ shows the smallest variation, especially compared with Rct and RSEI, and only slightly increases at higher temperatures or lower SOH, indicating that R_0_, which originates primarily from the electrolyte, electrode conductors, and internal connections, is not highly sensitive to battery aging. By contrast, RSEI and Rct increase more noticeably, particularly Rct, which demonstrates a clear upward trend as SOH drops from 100% to 80%. This suggests that, as the battery ages, the electrochemical activity at the electrode–electrolyte interface declines, electron transfer becomes more hindered, and interfacial polarization increases. Moreover, under high SOC and stable temperature, impedance values remain relatively low, indicating high interfacial activity and better electrochemical performance.

Figure 10a–c illustrate the variations in the three impedance parameters (i.e., ohmic resistance (R_0_), SEI film resistance (RSEI), and charge transfer resistance (Rct)) of LIBs under different SOC conditions (i.e., SOC = 0%, 50%, and 90%) and different compression displacements. From the overall trend, it is evident that all three impedance parameters generally increase as the compression displacement increases (i.e., from 0 to 8 mm). This indicates that external mechanical compression damages the internal structural integrity of the battery, thereby affecting the transport pathways of electrons and ions.

When the SOC is at 0% (as shown in Figure 10a), the fitted impedance parameters also increase with compression displacement. In particular, Rct increases markedly under severe compression, indicating that the charge transfer process becomes strongly constrained under the combined effect of low SOC and mechanical deformation. When the SOC is at 50% (as shown in Figure 10b), R_0_ and Rct exhibit a steady increasing trend. The increase in Rct reflects serious disruption of electrode/electrolyte interfacial reactions, degraded contact, and blocked active sites. While RSEI remains at a relatively low level, it increases slightly, possibly due to cracks in the SEI film or uneven thickening caused by compression. When the SOC is at 90% (as shown in Figure 10c), the increasing trends of R_0_, Rct, and RSEI are most significant, particularly Rct. This indicates that, at high SOC, the internal stress of the battery is more concentrated, and electrode activity is stronger, making the electrochemical performance more vulnerable to mechanical damage.

**Figure 11:**
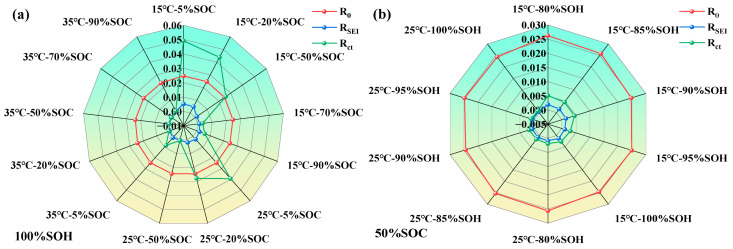
Variations of lithium-ion battery parameters under different states; (**a**) impedance characteristic parameters of LIBs under different temperatures and SOC levels; (**b**) impedance characteristic parameters of LIBs under different temperatures and SOHs.

To better understand the parameter variation patterns of LIBs ECM, we constructed radar charts of impedance parameter variations under different SOC, temperature, and SOH conditions (as shown in Figure 11).

From Figure 11, it can be further observed that the parameters of the LIB’s equivalent circuit model are influenced by the coupling effects of SOH, SOC, and temperature. As SOH decreases, the SEI film becomes increasingly unstable, and the interfacial reaction activity weakens, resulting in a general rise in both RSEI and Rct. Regarding SOC, both extremely high and low SOC levels tend to intensify polarization and side reactions, thereby increasing the impedance. In contrast, moderate SOC levels are most favorable for interfacial electrochemical processes. An increase in temperature generally leads to a reduction in impedance across all operating conditions, with the effect being most significant in the low-to-moderate SOC range, confirming the thermally activated nature of interfacial processes.

## 4. Conclusions

This study investigates the degradation mechanisms and failure characteristics of LIBs under various operating conditions based on EIS. An equivalent circuit model incorporating ohmic resistance (R_0_), SEI film resistance (RSEI), charge transfer resistance (Rct), and double-layer capacitance (CPE) was constructed. Multiple sets of controlled variable experiments were designed and conducted to measure and fit the impedance responses under varying temperature, mechanical compression displacement, SOC, and SOH. The main conclusions are as follows:

(1) Temperature variation significantly affects internal resistance and interfacial electrochemical kinetics. At low temperatures, ion migration slows down, resulting in increased R_0_ and Rct, severe system polarization, and pronounced dispersive behavior. Although elevated temperatures help reduce interfacial impedance, they may compromise SEI structural stability.
(2) As compressive displacement increases, the low-frequency region of the EIS shows pronounced distortion, accompanied by nonlinear increases in RSEI and Rct. These results indicate that mechanical compression significantly alters the impedance response and aggravates interfacial polarization and transport-related resistance. Therefore, mechanical stress is identified as an important influencing factor in electrochemical performance degradation under compressive loading.
(3) Batteries operating in a moderate SOC range (approximately 40–60%) exhibit lower Rct and relatively stable phase angle characteristics. In contrast, both high- and low-SOC conditions are associated with increased impedance and stronger polarization features. These results indicate that the impedance response shows a U-shaped dependence on SOC, with the most favorable electrochemical response observed in the intermediate SOC range.
(4) As SOH decreases, both RSEI and Rct increase progressively, indicating enhanced interfacial resistance and charge transfer limitation during aging. Notably, Rct remains relatively stable in the early aging stage but becomes more sensitive in the intermediate degradation stage, while RSEI shows a sustained upward trend throughout the tested SOH range. These parameters therefore provide useful indicators for impedance-based aging assessment.

Based on the multidimensional experimental results and the evolution patterns of impedance characteristics, it can be concluded that temperature, SOC, SOH, and mechanical compression all have significant influences on the impedance response of LIBs. Temperature and mechanical compression mainly affect the magnitude and distribution of impedance components, while SOC and SOH strongly influence interfacial polarization and diffusion-related behavior. The combined EIS, ECM, and DRT analyses provide a useful framework for identifying impedance evolution trends under different operating conditions. These findings support the application of EIS as a non-invasive tool for battery state monitoring and impedance-based health assessment. From an engineering perspective, the present results suggest that moderate SOC operation is more favorable for maintaining a lower impedance response, whereas low temperature, aging, and mechanical compression all aggravate impedance growth and polarization-related behavior. These findings indicate that, during battery assembly and module integration, excessive compressive loading should be avoided, and the cells should preferably be operated or stored within moderate SOC windows when possible. It should also be noted that the present conclusions were obtained from commercially available 18650 NCM lithium-ion cells. Therefore, the specific quantitative trends reported here are most directly applicable to this cell format and chemistry. For other battery chemistries or cell formats, the detailed impedance evolution behavior may differ because of differences in materials, structural design, and mechanical constraint conditions. Nevertheless, the combined EIS–ECM–DRT analytical framework developed in this work may still provide useful guidance for comparative state evaluation in other battery systems.

## Data Availability

The original contributions presented in this study are included in the article. Further inquiries can be directed to the corresponding author(s).
